# Collective Movement in the Tibetan Macaques (*Macaca thibetana*): Early Joiners Write the Rule of the Game

**DOI:** 10.1371/journal.pone.0127459

**Published:** 2015-05-20

**Authors:** Xi Wang, Lixing Sun, Jinhua Li, Dongpo Xia, Binghua Sun, Dao Zhang

**Affiliations:** 1 School of Resources and Environmental Engineering, Anhui University, Hefei, Anhui, China; 2 Department of Biological Sciences, Central Washington University, Ellensburg, Washington, United States of America; 3 School of Life Science, Anhui Normal University, Wuhu, Anhui, China; 4 School of Life Sciences, Anhui University, Hefei, Anhui, China; University of Pisa, ITALY

## Abstract

Collective behavior has recently attracted a great deal of interest in both natural and social sciences. While the role of leadership has been closely scrutinized, the rules used by joiners in collective decision making have received far less attention. Two main hypotheses have been proposed concerning these rules: mimetism and quorum. Mimetism predicts that individuals are increasingly likely to join collective behavior as the number of participants increases. It can be further divided into selective mimetism, where relationships among the participants affect the process, and anonymous mimetism, where no such effect exists. Quorum predicts that a collective behavior occurs when the number of participants reaches a threshold. To probe into which rule is used in collective decision making, we conducted a study on the joining process in a group of free-ranging Tibetan macaques (*Macaca thibetana*) in Huangshan, China using a combination of all-occurrence and focal animal sampling methods. Our results show that the earlier individuals joined movements, the more central a role they occupied among the joining network. We also found that when less than three adults participated in the first five minutes of the joining process, no entire group movement occurred subsequently. When the number of these early joiners ranged from three to six, selective mimetism was used. This means higher rank or closer social affiliation of early joiners could be among the factors of deciding whether to participate in movements by group members. When the number of early joiners reached or exceeded seven, which was the simple majority of the group studied, entire group movement always occurred, meaning that the quorum rule was used. Putting together, *Macaca thibetana* used a combination of selective mimetism and quorum, and early joiners played a key role in deciding which rule should be used.

## Introduction

Collective decision making has recently attracted a great deal of interest in both natural and social sciences. In collective movements, the decision to move requires a consensus between the initiator’s proposal and the acceptance of other members of the group [1]. Among large group living species, such a consensus may emerge from simple interaction rules based on local communication principles [2]. Social insects, for instance, often use simple and local rules among the insects themselves and between the insects and their environment to coordinate collective movements [3,4]. Recent studies have demonstrated that such self-organized processes can also exist in small groups with global communication [5]. Many of such studies have focused on the role of initiator (e.g. dwarf mongooses, *Helogale undulata* [6], Prezwalski horses, *Equus ferus* [7], brown lemurs, *Eulemur fulvus* [8], white-faced capuchins, *Cebus capucinus* [9], and human election [10]). Only a few species, however, have been examined to answer how members decide to join the group activities [5,11,12]. Since joining is an indispensable step prior to or during any collective movement, it plays a key role in social coordination. Therefore, knowing the rules used in the joining process is crucial for understanding how collective decisions are made in animal and human societies.

Mimetism is often hypothesized as a self-organized rule used during the joining process. It has two types: 1) anonymous mimetism, where the probability of an individual joining a collective movement depends on the number of individuals already in the group, regardless of their identities [1], and 2) selective mimetism, where the probability of an individual joining a collective movement depends on its social relationships with the members already in the group [5]. In mammals living in small groups, although anonymous mimetism during collective movements has been observed in species such as merino sheep (*Ovis aries*) [13] and white-faced capuchins [14], it appears less common than selective mimetism, which has been found in a wider array of species such as Indian palm squirrel (*Funambulus pennanti*) [15], rhesus macaques (*Macaca mulatta*) [16], Tonkean macaques (*M*. *tonkeana*) [5], free-ranging dogs (*Canis lupus*) [17], and domestic geese (*Anser domesticus*) [18]. Because being highly associated with related or dominant individuals may increase an individual’s fitness, affiliative relationship, a key feature in selective mimetism, can prompt individuals to join collective movements [11,19]. Apparently, the fitness advantage of coordinated activities among affiliated individuals explains why selective mimetism as a rule used in the process of collective movements [11,17,19,20].

Mimetic behavior, however, is sometimes insufficient to explain non-linear response from individuals to those already participating in the group movement. In these situations, quorum rules are invoked as an alternative to mimetism. According to Conradt and Roper [21], a quorum refers to the minimum number of group members required to take or favor a particular action for the whole group to adopt this action. A response to a quorum is observed when the probability of members exhibiting a particular behavior depends on the number of individuals already performing the behavior [22–25]. Quorum has been shown an important mechanism in decision making among ants (*Temnothorax*) [22], honey bees (*Apis mellifera*) [23], three-spine sticklebacks (*Gasterosteus aculeatus*) [25], Tonkean macaques [26] and most common of all, humans [27,28]. For instance, Petit et al. [1] found that in white-faced capuchins, the whole group has a high probability of moving when at least four monkeys move in the same direction, indicating that a quorum rule is used in the process. In hamadryas baboons (*Papio hamadryas*), group members decide whether to follow male initiators, and during the decision-making process, the entire troop goes in the direction taken by the majority of group members [29]. Furthermore, Sueur et al. [26] provide quantitative evidence that similar quorum processes exist in Tonkean macaques. Unfortunately, quantitative data are still far from sufficient to ascertain whether group members truly decide to join movements according to quorum rules [30].

Although many species are capable of inter-individual recognition and/or display stable relationships among group members [31], few studies have probed into how joining decisions are related to the structure of a social network [5,32]. In a recent study, Sueur and Petit [11] applied social network metrics to their analysis of collective movements, showing that, based on the resulting association patterns, one can determine if a population is divided into subgroups, if the strength of association differs between individuals, or if some individuals play a more central role in group cohesion than others [33–35]. Therefore, using network metrics is an efficient tool to assess which rules may underlie the joining process during collective movements.

In this study, we used social network analysis to investigate the joining process in a small group of Tibetan macaques (*M*. *thibetana*), whose movements regularly switch from the feeding site to nearby forest. The Tibetan macaque is classified as near threatened by the IUCN and is listed on appendix II of the CITES list. Tibetan macaques are highly gregarious. Group members know each other and are familiar with their environment via global communication principles [36]. Females remain in their natal groups throughout their lives, whereas males disperse from their natal groups when they mature [37,38]. Tibetan macaques demonstrate a despotic dominance style, exhibiting low rates of counter-aggression and low conciliatory tendencies [37]. Frequent group movements have been observed in this species. The joining rule used in group movements, however, remains unknown. Here, we tested the two hypotheses of quorum versus mimetism. Quorum predicts the existence of a threshold that can make the whole group move together all the time, whereas mimetism does not predict such a threshold even though it may also result in whole group movements. Furthermore, we tested selective mimetism against anonymous mimetism, if mimetism was indeed used in the joining process. Selective mimetism predicts that participants are unequal in attracting others to join collective movements whereas anonymous mimetism lacks this feature. To test anonymous versus selective mimetism, we systematically screened for the influences of common individual attributes such as sex, rank, age, and social affiliation on joiners in collective movements.

## Materials and Methods

### Ethics Statement

This study complies with the regulations of the Chinese Wildlife Conservation Association regarding the ethical treatment of research subjects, and under the law of People’s Republic of China on the protection of wildlife. The study was fully observational, and our data collection did not affect the monkeys’ welfare. Huangshan Monkey Management Center and the Huangshan Garden Forest Bureau permitted us to conduct research at the field site.

### Study Site and Subjects

The study was conducted from August to December of 2012 at Mt. Huangshan National Reserve located in Anhui province, China. The reserve is a UNESCO World Culture and Nature Heritage site as well as a well-known tourist destination [39]. The study site is publicly owned. Similar to other macaques, Tibetan macaques display linear dominance hierarchies [37].

The group of Tibetan macaques in our field study was known as Yulinkeng 1 (YA1), which had been continually observed since 1986. At the time of our research, the troop constituted a total of 32 members including four adult males, eight adult females, six sub-adults, nine juveniles, and five infants. YA1 inhabits an area within the reserve known as the “Valley of the Wild Monkeys” (N30° 04’ 25.1” / E118° 08’ 59.3”) [40]. This area is characterized by steep, mountainous terrain. The group of monkeys were wild. They engaged in social activities in nearby forest during most of the day without any restriction on their home range. For the convenience of viewing by tourists, they were supplied with 3–4 kilograms of corn daily [39–41]. After corn feeding, they regularly switched locations from the feeding site to forest. Collective movements often occurred at the time of the switch.

Our focal animals were the 12 adults of YA1, which had been habituated to human presence. They were individually recognized based on distinctive physical features such as scars, hair color patterns, or facial/body appearances [36,39]. Prior studies have provided detailed information about individual identities and life histories for all the members [40]. Key biological attributes in terms of sex, hierarchical rank and age of the 12 adults studied are provided in Table 1.

**Table 1:** Attributes of focal animals in YA1 during observation.

Individuals	Sex	Rank	Age	Focal Duration(sec.)	Individuals	Sex	Rank	Age	Focal Duration(sec.)
TG	Male	1	9	55200	YH	Female	1	9	55200
ZL	Male	2	12^a^	55200	Hhui	Female	2	7	54600
GS	Male	3	28	54600	YM	Female	3	22	55800
BT	Male	4	20^a^	55800	TH	Female	4	9	55800
					HH	Female	5	9	55800
					TR	Female	6	8	55200
					TT	Female	7	21	55200
					YZ	Female	8	20	54600

^a^These two individuals were immigrants from other groups. Their ages were estimated based on physical features [36].

### Definitions and Behavioral Observations

We observed the focal group seven hours per day from 08:30 to 11:30 and from 13:30 to 17:30. We recorded collective movements via a digital video camera (Canon EOS 550D). Based on our preliminary observation for YA1 (August 1^st^-14^th^, 2012), we used a set of criteria similar to those used by Sueur and Petit [16] for collecting data about collective movements so that our results are comparable with existing macaque literature. The following are operational definitions for the key terms used in our study.

- Initiation: starting when the first adult walks more than 10 meters in less than 30 seconds. This criterion allowed us to discriminate between the initiation of a collective movement and other movements such as feeding movements. Sub-adults, juveniles, and infants were excluded from initiators because they never incited any entire group movement during our preliminary observation.
- Joiner: any individual that walks more than five meters within 45° in the direction to which the initiator departs before the joining is terminated [16]. A joiner that moves in the first five minutes after the initiator departs is defined as an early joiner. For the convenience of presentation, initiators were also counted as earlier joiners. The criterion of five minutes is the minimum duration that can result in an entire group movement based on our preliminary observation. In our data analyses, an initiator was also considered as an early joiner because, by definition, it participated in movements within the first five minutes. More importantly, this broader definition allowed us to focus on the role of all early joiners in collective movements. Since our study focused on adults, early joiners referred exclusively to adults accordingly.
- Termination of joining: when no more individual joins the movement within five minutes after the departure of the first individual or after the joining of the last individual [16]. That is, the joining process is considered finished when the delay of the next individual joining the movement exceeds five minutes.
- Entire group movement: a collective movement that has at least two-thirds of all the group members joining the movement before termination. During the mating season, we observed 171 entire group movements initiated by 12 adult members. The duration of an entire group movement was 9.60±3.53 (mean ± SD) minutes.

**Fig 1:**
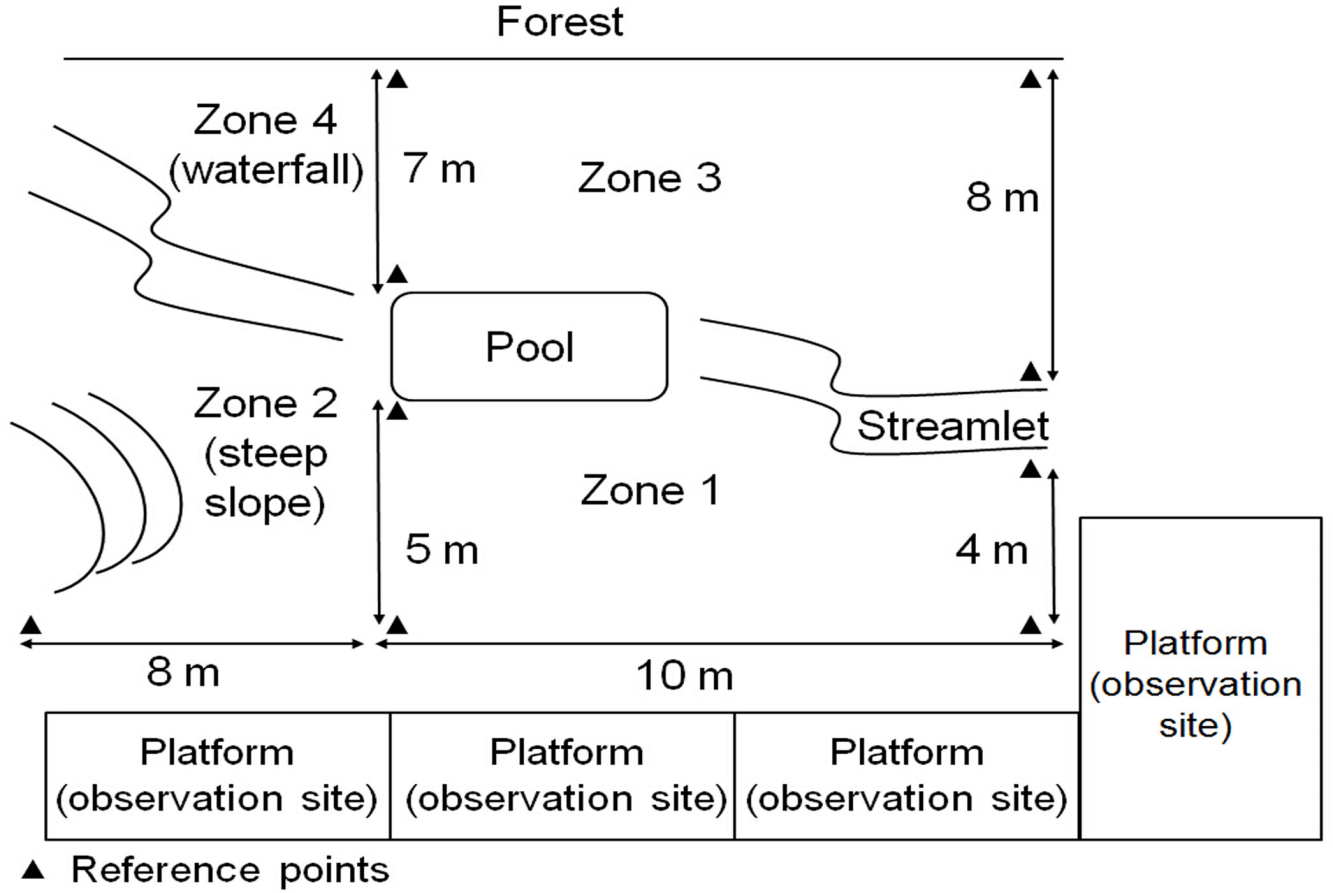
Diagram of feeding site for group YA1.

The feeding site was marked with a systematic grid of reference points and divided into four zones (Fig 1). This allowed us to accurately record the positions and movement distances for each animal. The starting zone refers to the area in feeding site where less than or equal to 10 meters from the starting point of initiator [16]. We included movement events only if at least two-thirds of the group members were present in the starting zone when they occurred [32].

Measurements during collective movements were taken using the all occurrence sampling method [42].

### Data Analyses

To quantify how often two early joiners occur together in collective movements, we used the half-weight index (HWI) [43] calculated by the number of two individuals seen together divided by the total number of movements. Its value ranges between 0 (never associated) and 1 (always associated) [11]. We drew the topology of co-occurrence for early joiners during movements via Netdraw in UCINET 6.0 [44].

We calculated eigenvector centrality coefficient via HWI matrices using SOCPROG 2.4 [45–49]. Eigenvector centrality coefficient measures how closely associated an individual is to others in collective movements. A high value means either that the individual is connected to more group members than otherwise or that the individual is connected to others that are also highly central in collective movements [46]. Based on eigenvector centrality coefficient, we quantified the attraction of every early joiner to others during joining processes.

To affirm the importance of early joiners on the joining process, we analyzed the correlation between the joining position of every joiner and its centrality coefficient. The joining order index was calculated for each individual per group movement with the following formula: 1－[I－1/N－1], where I is the position in the order of group progression taken by the individual, and N is the number of group members [50]. The index ranges from 1 (= first position) to 0 (= last position). We scored each individual's mean joining order index using this formula.

To evaluate whether affiliative relationship influences the joining process in collective movements, we correlated the co-occurrence of early joiners in collective movements with that in other daily group activities measured by proximity. To do so, we calculated the eigenvector centrality coefficients based on HWI and the dyadic association index (DAI). DAI measures how frequently two individuals X and Y are associated during their daily group activities except collective movements [51]: D_ab_/(D_a_+D_b_-D_ab_), where D_ab_ refers to the duration in which X and Y are seen within one meter of each other, D_a_ refers to the duration when X is seen, and D_b_ refers to the duration when Y is seen. The DAI centrality coefficient quantifies the attraction of an individual to other group members during group activities other than collective movements.

To obtain durations data for calculating DAI, we used focal animal sampling and continuous recording via a digital voice recorder [42]. We used 10 minutes as the duration of each focal sample so that all adults could be sampled at least once a day (Table 1) [52].

To assess the effect of early joiners’ social ranks on the joining process, we determined the dominance ranks of the 12 adults by aggressive and submissive interactions using the event behavior sampling method [37,42,52]. Aggressive interactions were scored when one individual stared, hit, chased, or scratched another individual [36]. Submissive interactions include such behaviors as fearful grin, cower, mock leave, avoid, flee, or scream during social interactions [37]. We considered an individual in a lower rank if it displayed submissive behavior toward another group member. On the contrary, the individual to which a lower ranking member submitted was considered in a higher rank [36,52].

### Statistical Analyses

To analyze the effect of sex on the joining process and to evaluate the attraction differences of early joiners in the social network, we used independent-samples t test for situations involving two samples and K-independent-samples Kruskal-Wallis test for situations involving three or more samples. To test the effect of age and rank on the joining process, the relationship between the joining order of a joiner and its centrality coefficient, and to examine the effect of social affiliation on the joining process, we used Spearman rank correlation analysis. Since t test is parametric, we used one-sample Kolmogorov-Smirnov test to assure that the normality assumption of sample distribution was not violated. All tests were conducted using SPSS (version 13.0), and the level of significance was set at 0.05 a priori.

## Results

**Fig 2:**
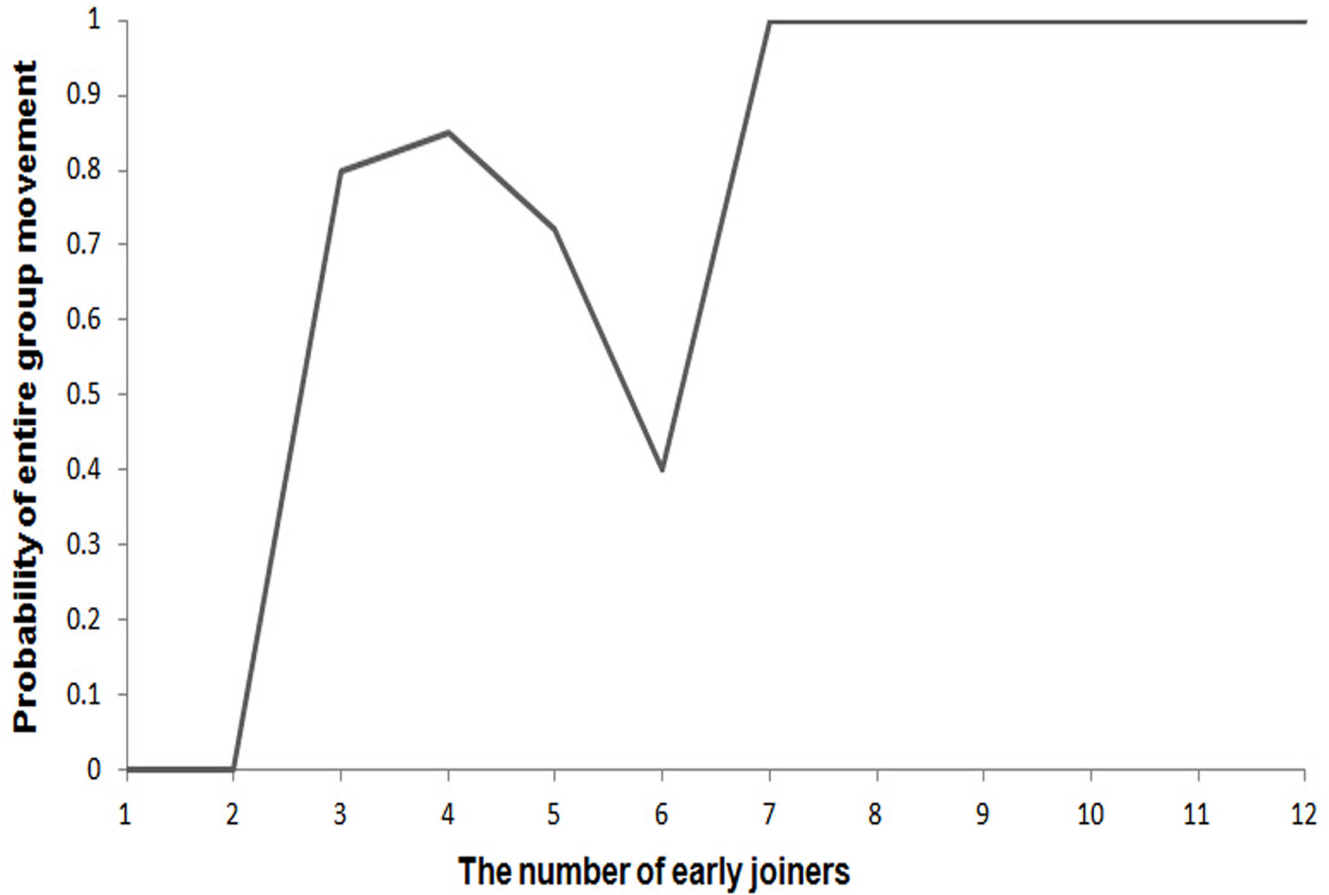
Relationship between the number of early joiners and the probability of collective movement by the entire group.

To find a threshold that might trigger the departure of the entire group, we assessed the relationship between the number of early joiners and the probability of entire group movement (Fig 2). We found that, when less than three early joiners (all adults) participated in movements during the first five minutes of the joining process, no entire group movement occurred before the joining process is terminated. When the number of early joiners ranged from three to six, the probability of the entire group response fluctuated between 40% to 85% without a consistent pattern. Nonetheless, once the threshold of seven was reached, the probability of entire group movement became 100%. In other words, when equal to or more than seven adults participated in movements during the first five minutes of the joining process, entire group movement always occurred.

**Fig 3:**
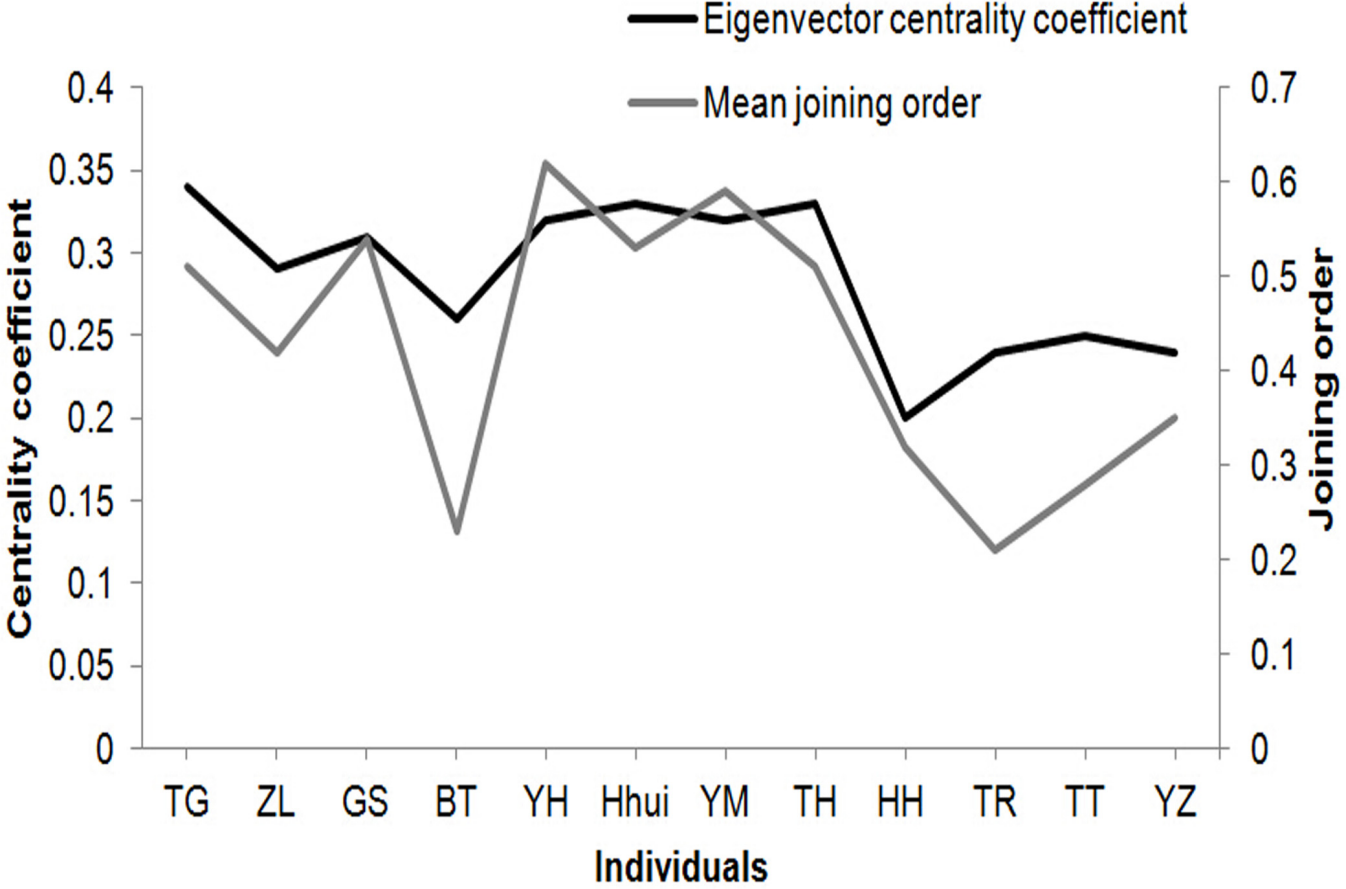
Relationship between joining order and eigenvector centrality coefficient. Individuals of the same sex are presented from left to right (males for the first four and females for the next eight) in the descending order in hierarchy.

To validate whether early joiners played an important role on joining processes, we analyzed the correlation between the mean joining position and eigenvector centrality coefficient for every adult macaque in the group (Fig 3). We found a positive correlation (Spearman rank correlation r_s_ = 0.695, N = 12, *P*<0.05).

**Fig 4:**
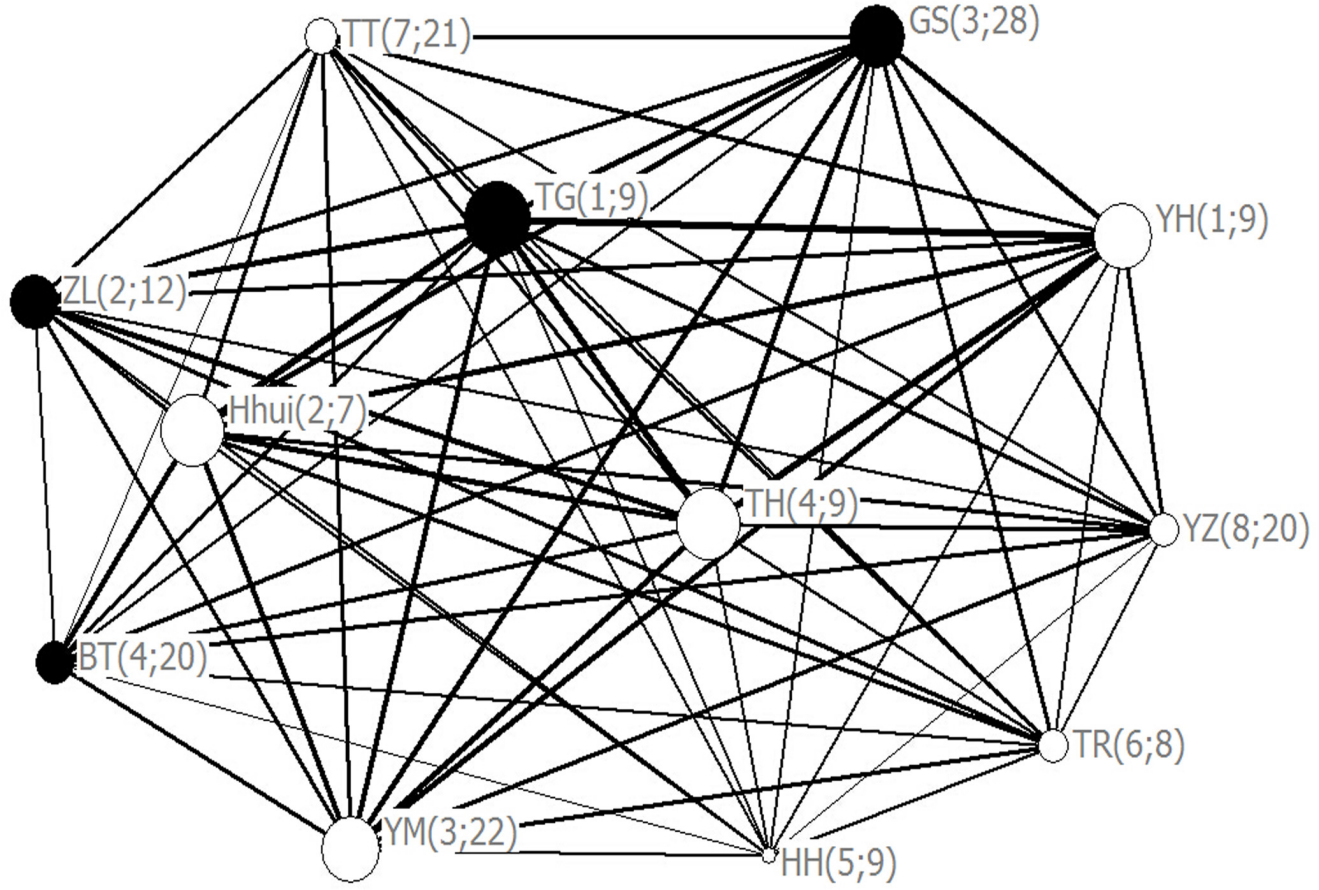
Eigenvector centrality coefficients of early joiners in social network. Black circles represent males and white circles represent females. The numbers inside each pair of brackets indicate social rank and age, respectively. Line thickness is proportional to HWI value, and the size of a node to the value of eigenvector centrality coefficient.

To answer why the response of the entire group fluctuated when the number of early joiners ranged from three to six (see Fig 2), we explored key attributes of early joiners in the social network. Results show that early joiners differed significantly in eigenvector centrality coefficient (Kruskal-Wallis test: df = 11, *P*<0.05, Fig 4). The difference between adult males and females was insignificant (t = 0.738, df = 10, *P*>0.05). Also, age and eigenvector centrality coefficient were not correlated (Spearman rank correlation r_s_ = –0.174, N = 12, *P*>0.05). However, social rank was positively correlated with eigenvector centrality coefficient in both adult males (r_s_ = 0.800, N = 4, *P*<0.05) and females (r_s_ = 0.655, N = 8, *P*<0.05).

**Fig 5:**
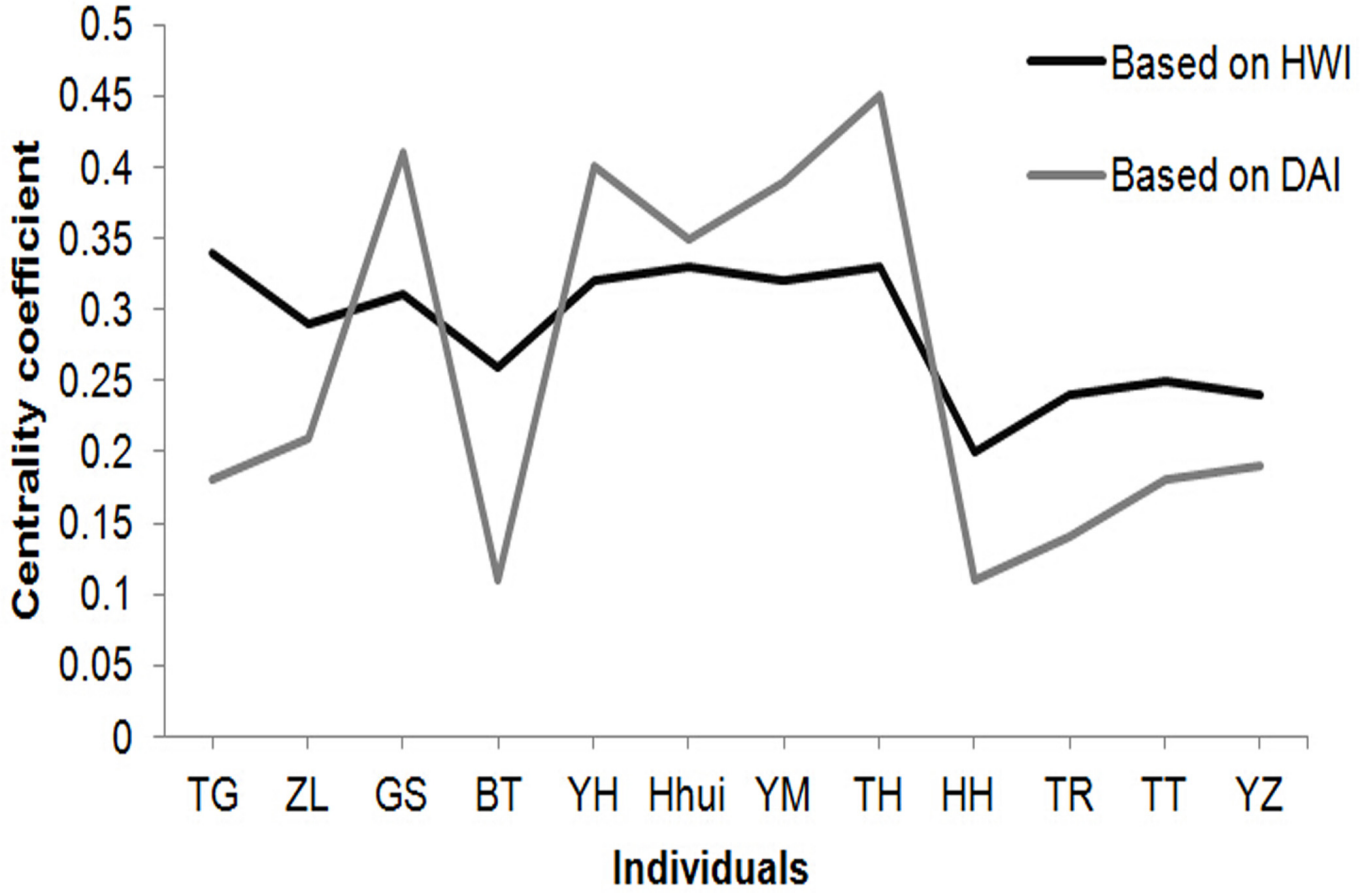
Eigenvector centrality coefficients of individuals based on HWI and DAI. Individuals of the same sex are presented from left to right (males for the first four and females for the next eight) in the descending order in hierarchy.

Finally, to analyze the effect of social affiliation of early joiners on the joining process, we compared the eigenvector centrality coefficients of individuals based on HWI and DAI, characterizing, respectively, when subjects were in collective movements (i.e. co-occurrence) and when they were engaged in other daily activities (i.e. proximity). We found a positive correlation between the two coefficients (Spearman rank r_s_ = 0.614, N = 12, *P*<0.05, Fig 5).

## Discussion

Several of our findings were interesting. First, our results show that early joiners, when their numbers ranged between three and six, could elicit varied response from other group members as to whether or not to join the collective movement. Apparently, some factors of early joiners influenced the joining decision of others. We found that the more earlier a group member participated in a movement, the more important role it played during collective decision making, as shown by the positive correlation between mean joining order of every joiner and its eigenvector centrality coefficient. The latter index indicates how important the individual acts in joining network [11]. This result is comparable with situations in black howler monkeys (*Alouatta pigra*), where females at the front of a group movement have the highest centrality eigenvectors among the adult group members [53]. Our results indicate that the individual at the first position of a moving group (i.e. initiator) was not always the only decision maker. Early joiners could also play an important role during group movements, a result consistent with other primate species such as white-faced capuchin monkeys and African baboons [54,55].

Second, according to the social network graph, we found that higher-ranking early joiners had higher eigenvector centrality coefficients. Because eigenvector centrality coefficient can quantify the attraction of early joiners to other members during the joining process [46], the result means that higher-ranking early joiners were connected to more group members and were also highly central in collective movements. Similarly, alpha males have been reported to be the consistent decision makers in group movements in mountain gorillas (*Gorilla gorilla*) [56] and dominant individuals are more likely than subordinates to instigate movements to new foraging sites in green woodhoopoes (*Phoeniculus purpureus*) [57]. In all of these cases, the central individuals, which are in the front of the movements, seem to greatly influence the joining decision of other group members [6,33].

Also, we found that social affiliation could influence the joining process as well because early joiners who had higher centrality coefficients in daily activities also had higher centrality coefficients during collective movements. This means that early joiners with closer social affiliations could also attract more members during the joining process. This result is comparable to the findings in several other species such as Indian palm squirrel [15], free-ranging dogs [17], and chacma baboons (*Papio ursinus*) [58].

In our study, because both social rank of an early joiner and affiliation between group members affected the attraction of the early joiner to other members during the joining process, selective mimetism, rather than anonymous mimetism, was used as the joining rule in Tibetan macaques. Apart from our study, the use of selective mimetism has also been found in several other species [4,13,24,59,60]. In Tonkean macaques, for instance, how an individual decides to join a collective movement depends on whether its strongly affiliated individuals depart [5].

Some researchers suggest that in chacma baboons [58] and free-ranging dogs [17], long-term benefits of affiliative relationships with dominant leaders may include increased protection from predators and from infanticidal males [58]. This is because, in groups, low ranking or poorly affiliated individuals are typically peripheral with few and weak relationships with their conspecifics, whereas dominant and/or highly affiliated individuals interact with others more often [61]. In our study, it appears that early joiners who had higher social ranks and more frequent social interactions with others might be more attractive to members in the joining process than those of lower ranks and interacting less with others.

Selective mimetism may be explained by social styles [62]. For example, Tonkean macaques show an egalitarian social structure [11]. Individuals participate in a voting process (quorum) prior to collective movements, the lack of centrality for dominant or old Tonkean macaques suggests that all individuals may have equal weight in the voting process and interactions are not constrained by individual status in the species [11]. However, rhesus macaques, with a more pronounced dominance hierarchy, prefer to join high-ranking or related individuals during collective movements (selective mimetism) [11,16,32]. High-ranking individuals have high centrality coefficients and thus are more attractive to other members than are low-ranking individuals during the joining process [11]. Tibetan macaques demonstrate a despotic dominance style [37]. In our study, higher-ranking early joiners were connected to a larger number of individuals than were lower-ranking group members, not only in collective movements but also during other daily social activities. This agrees with the study on rhesus macaques which have a similar social structure [11].

Our study also shows that when the number of early joiners accumulated to seven or more, the entire group would participate in collective movement all the time. Clearly, this threshold of seven indicates the use of quorum as the joining rule during collective movements in Tibetan macaques. Quorum rules appear to be more common for self-organization in large groups [31]. In the quorum process of shoaling [25], for instance, three-spine sticklebacks exhibit a highly non-linear response to their immediate neighbors. While largely disregarding the movement of a strange member, sticklebacks tend to follow neighbors committed to a given direction of travel [25]. Though somehow surprising, the use of the quorum rule in the small group of the Tibetan macaques we studied may be explained by the reduction in risk of getting lost from groups. Living in groups offers a number of benefits for individuals including reduced per capita predation risk through shared vigilance or predator confusion [63] in addition to opportunities to cooperate with kin [64]. To reap the benefit of group living, it requires that, when the number of joiners in a collective decision has reached a certain level, all others have to follow the decision no matter whether or not it conflicts with their best individual interests. Such quorum as the joining rule is also found in white-faced capuchins, rhesus macaques, and Tonkean macaques, all of which use the threshold of four in collective movements [1,12]. Our findings provided quantitative evidence for a similar process in Tibetan macaques. They again affirm the use of quorum rules in collective decision making in small, close-knit groups.

In conclusion, our study led to two surprising findings. First, Tibetan macaques used a combination of quorum and selective mimetism in collective decision making. Second, the number of early joiners played a critical role as to which rule was used. In our study, the threshold of seven early joiners as the quorum rule for entire group movements exactly exceeds the half number of adult members. It agrees with the majority rule among the adults. Since threshold tends to vary with group size [65], we are uncertain whether the threshold found in our study was truly based on the majority rule or by coincidence. Future studies are needed to test whether this simple majority rule still holds for the decision making process in Tibetan macaques with varying group sizes and in other species. This will lead us to a better understanding as to whether a universal pattern exists for group coordination through collective decision making.
